# Controlled Metal–Support Interactions in Au/CeO_2_–Mg(OH)_2_ Catalysts Activating the Direct Oxidative Esterification of Methacrolein with Methanol to Methyl Methacrylate

**DOI:** 10.3390/nano11113146

**Published:** 2021-11-21

**Authors:** Nagyeong Kim, Seulgi Lim, Seungdon Kwon, Yuyeol Choi, Ji-Woong Lee, Kyungsu Na

**Affiliations:** 1 Department of Chemistry, Chonnam National University, Gwangju 61186, Korea; brightest24@gmail.com (N.K.); swing9sg@gmail.com (S.L.); kwon950515@gmail.com (S.K.); chi3072@gmail.com (Y.C.); 2 Department of Chemistry, University of Copenhagen, 2100 Copenhagen, Denmark; jiwoong.lee@chem.ku.dk

**Keywords:** oxidative esterification, methacrolein, methyl methacrylate, Au catalyst, strong metal–support interaction, calcination

## Abstract

The strong metal–support interaction (SMSI) between the three components in Au/CeO_2_–Mg(OH)_2_ can be controlled by the relative composition of CeO_2_ and Mg(OH)_2_ and by the calcination temperature for the direct oxidative esterification of methacrolein (MACR) with methanol to methyl methacrylate (MMA). The composition ratio of CeO_2_ and Mg(OH)_2_ in the catalyst affects the catalytic performance dramatically. An Au/CeO_2_ catalyst without Mg(OH)_2_ esterified MACR to a hemiacetal species without MMA production, which confirmed that Mg(OH)_2_ is a prerequisite for successful oxidative esterification. When Au/Mg(OH)_2_ was used without CeO_2_, the direct oxidative esterification of MACR was successful and produced MMA, the desired product. However, the MMA selectivity was much lower (72.5%) than that with Au/CeO_2_–Mg(OH)_2_ catalysts, which have an MMA selectivity of 93.9–99.8%, depending on the relative composition of CeO_2_ and Mg(OH)_2_. In addition, depending on the calcination temperature, the crystallinity of the CeO_2_–Mg(OH)_2_ and the surface acidity/basicity can be remarkably changed. Consequently, the Au-nanoparticle-supported catalysts exhibited different MACR conversions and MMA selectivities. The catalytic behavior can be explained by the different metal–support interactions between the three components depending on the composition ratio of CeO_2_ and Mg(OH)_2_ and the calcination temperature. These differences were evidenced by X-ray diffraction, X-ray photoelectron spectroscopy, and CO_2_ temperature-programmed desorption. The present study provides new insights into the design of SMSI-induced supported metal catalysts for the development of multifunctional heterogeneous catalysts.

## 1. Introduction

The design of multifunctional catalysts has long been investigated with the aim of minimizing complicated multistep reaction processes and increasing reaction efficiency [1,2,3,4]. In heterogeneous catalysts, various catalytic functions can be simultaneously introduced to a single catalyst architecture by various synthetic methods [1,2,3,4,5,6]. The simple addition of active components to a synthesis mixture can generate multifunctional catalytic sites [5,6,7], and the resultant multifunctional catalysts can reduce the number of reaction steps and, thereby, save on the overall energy consumption [2,3,4]. As well as allowing a more ecofriendly chemical process, multifunctional catalysts often possess unexpected catalytic functions derived from synergistic electronic interactions between catalytic components in proximity [5,6,7]. Strong metal–support interactions (SMSIs), which are unique electronic interactions at the interface between the catalytic metal nanoparticles and the supporting materials (typically, nanoporous metal oxides), are a representative example of unexpected synergies [7,8,9].

The SMSI effect can not only enhance catalytic activity but can also control product selectivity [10,11,12]. For example, with specific metal nanoparticles as the main catalytic site, variation of the metal oxide support can change the catalytic behavior [13,14]. Conversely, with a given metal oxide support, variation of the metal nanoparticles can also change the catalytic behavior [15,16,17]. In general, SMSIs change the surface characteristics of the main metal nanoparticles and the metal oxide supports, such that the electronic properties of the catalyst surface can be changed [18,19,20]. This unique effect results in changes to the interaction of the catalyst surface with the approaching reactant molecules and their adsorption geometries, the formation of intermediate species and transition states, and finally the thermodynamics and kinetics of product formation [14,15]. Therefore, multifunctional catalysts are important, not only in terms of reaction efficiency but also in terms of the generation of SMSI effects to induce unexpected catalytic behavior [16,17,18].

The direct oxidative esterification (DOE) of aldehydes reacting with alcohols to produce alkyl esters is a green chemical route for the production of valuable alkyl esters via a one-pot single-step process [20,21,22,23,24,25,26]. This reaction can utilize oxygen or air as a green oxidant, and a multifunctional catalyst is a prerequisite for guiding a desirable reaction pathway. Recently, we reported Au nanoparticles supported on CeO_2_–Mg(OH)_2_ as a multifunctional heterogeneous catalyst with an SMSI at the interfaces of the three catalytic components (i.e., Au, CeO_2_, and Mg(OH)_2_). The catalyst exhibited high catalytic activity for aldehyde conversion and product selectivity for a desirable alkyl ester [7]. Following the published work, we carried out in-depth studies of the SMSI’s effects on the catalytic behavior during DOE reactions of methacrolein (MACR) with methanol to produce methyl methacrylate (MMA), by controlling two important synthetic parameters during catalyst preparation. First, the composition of CeO_2_ and Mg(OH)_2_ in the catalysts was systematically controlled in the Au/CeO_2_–Mg(OH)_2_ catalysts. Second, the crystallinities of the Au/CeO_2_–Mg(OH)_2_ catalysts were controlled by variation of the calcination temperature. Comprehensive characterizations and reaction studies revealed that the control of these two synthetic parameters changed the structural properties of the resultant catalysts, and consequently, the reaction behaviors were remarkably changed. In addition, structure–activity relationships were derived for a fundamental understanding of the catalytic behavior of Au/CeO_2_–Mg(OH)_2_ as a multifunctional catalyst. This understanding opens up the possibility of developing high-performance catalysts.

## 2. Materials and Methods

### 2.1. Preparation of Materials

The materials investigated in this work were synthesized by referring to the work published recently by the same authors [7]. Various CeO_2_–Mg(OH)_2_ supports with different compositions were prepared by controlling the molar composition of the Ce and Mg precursor with ratios of 100:0, 90:10, 80:20, 70:30, 60:40, 50:50, 37:63, 20:80, 10:90, and 0:100. In a typical synthesis, Ce(NO_3_)_3_·6H_2_O (Daejung, Siheung, Korea) and Mg(NO_3_)_2_·6H_2_O (Daejung) were dissolved together in distilled water to make a solution containing the metal precursor with H_2_O/metal = 65; citric acid monohydrate was dissolved in the solution with citric acid monohydrate/metal = 1, which was stirred at 80 °C for 5 h and subsequently dried in an oven at 100 °C overnight. The resultant polymeric mixture was collected, ground to a fine powder, and calcined at 750 °C for 9 h under air flow to produce CeO_2_–MgO, which is denoted as CM. For the preparation of supported Au nanoparticle samples, distilled water containing 3 wt.% of HAuCl_4_ (2.55 × 10^−3^ M) was added dropwise to the solution containing CeO_2_–MgO under vigorous stirring. Then, it was stirred at 65 °C for 3 h, and the precipitated sample was subsequently filtered and washed with distilled water to wash any excess chloride anions. The resultant supported Au nanoparticle samples were dried at 60 °C for 5 h and are denoted as AuCM.

For the preparation of CeO_2_–Mg(OH)_2_ supports with different crystallinities, CM samples with a Ce:Mg ratio of 37:63 were calcined at temperatures of 450, 600, 750, and 1000 °C. These samples are denoted as CM(450), CM(600), CM(750), and CM(1000), respectively, according to the calcination temperature.

### 2.2. Characterization of Materials

XRD patterns were obtained by using a Rigaku MiniFlex 600 apparatus using Cu Kα radiation (λ = 0.1541 nm) at 40 kV and 15 mA (600 W). The measurements were recorded with a step size of 0.02°, scanning rate of 4° min^−1^, and 2θ range of 15°–70°. The Au content in the catalysts was determined with inductively coupled plasma–optical emission spectroscopy (ICP–OES; Avio 500, PerkinElmer, Waltham, MA, USA). N_2_ adsorption/desorption isotherms were measured by using a Micromeritics ASAP 2020 volumetric analyzer at liquid N_2_ temperature. All samples were degassed at 300 °C for 3 h prior to measurements. The surface areas were derived by using Brunauer–Emmett–Teller (BET) theory [27], and the total pore volumes and pore size distributions were obtained from the adsorption branches by using the Barrett–Joyner–Halenda (BJH) algorithm [28].

Transmission electron microscopy (TEM) images were obtained by using a JEM-2100F (JEOL, Tokyo, Japan) instrument operating at 200 kV (lattice resolution; 0.14 nm). X-ray photoelectron spectroscopy (XPS) was performed by using a K-ALPHA^+^ (Thermo Fisher Scientific, Waltham, MA, USA) equipped with a monochromatic Al Kα source connected to a 128-channel detector. For measurements, all samples were reduced under H_2_ and Ar flows (both at a rate of 20 mL min^−1^) at 300 °C for 5 h (ramping rate = 2.5 °C min^−1^). The spectra were fitted by using Gaussian–Lorentzian curves after baseline correction. CO_2_ temperature-programmed desorption–mass spectrometry (TPD–MS) was performed by using a BEL-CAT analyzer. The sample was first heated to 300 °C under a He flow, maintained at that temperature for 1 h, cooled to 40 °C, and then 5% CO_2_/He mixture flow was introduced for 1 h. After CO_2_ adsorption, the sample was flushed under a He flow to eliminate weakly physisorbed CO_2_, and the CO_2_ TPD–MS profile was obtained under a He flow with increasing the temperature from 50 to 600 °C.

### 2.3. Reaction Studies

The catalytic reaction was investigated by using a Teflon-lined stainless-steel autoclave. The Au-nanoparticle-supported catalyst was first reduced under H_2_ and Ar flows (both at a rate of 20 mL min^−1^) at 300 °C for 5 h (ramping rate = 2.5 °C min^−1^). For the reaction studies, 5.68 mL (0.0653 mol) of MACR (Antai, >95%) were dissolved in 14.67 mL of methyl alcohol (Daejung) (MACR:MeOH = 1:5) containing 1.5 g of the reduced catalyst. The reactor was purged with O_2_ to make the reactor saturated with O_2_, pressurized with O_2_ to 9 bar, then the temperature of the reactor was increased to 80 °C under magnetic stirring at 600 rpm. After the reaction was finished, the reactor was cooled in an ice bath, and the solid catalyst was separated from the reaction solution by using a syringe filter. The conversion and selectivity were analyzed with a gas chromatograph (Younglin YL6500, Anyang, Korea) equipped with a flame ionization detector and a capillary column (DB-5; length: 30 m; diameter: 0.32 mm; thickness: 1.5 μm) with ethanol as an internal standard.

## 3. Results and Discussion

### 3.1. Effect of Composition in CeO_2_–Mg(OH)_2_ Supporting Au Nanoparticles

**Figure 1:**
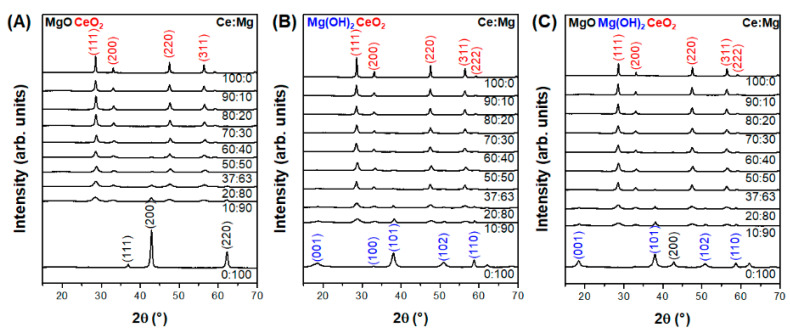
X-ray diffraction patterns of (**A**) as-synthesized CeO_2_–Mg(OH)_2_ with different Ce:Mg ratios in the synthesis gel, (**B**) Au-supporting CeO_2_–Mg(OH)_2_ before H_2_ treatment, and (**C**) Au-supporting CeO_2_–Mg(OH)_2_ after H_2_ treatment. The CeO_2_–Mg(OH)_2_ supports with different Ce:Mg ratios were calcined at 750 °C before supporting Au nanoparticles. The Miller indices in black, blue, and red correspond to MgO, Mg(OH)_2_, and CeO_2_, respectively.

Control of the gel composition for the synthesis of CeO_2_–Mg(OH)_2_ as the support for Au metal nanoparticles changed the framework composition in the resultant CeO_2_–Mg(OH)_2_, as evidenced by XRD patterns after calcination at 750 °C (Figure 1A). Pure MgO without the addition of CeO_2_ (Ce:Mg = 0:100) exhibited distinct XRD reflections corresponding to a cubic lattice with (111), (200), and (220) planes (*hkl* indices in black in Figure 1A) [29]. As the amount of Ce precursor increased in the synthesis gel composition, with a corresponding decrease in Mg precursor, the XRD peak intensities for MgO decreased gradually, and the XRD reflections corresponding to the cubic structure of CeO_2_, with different lattice parameters, became more prominent (*hkl* indices in red in Figure 1A) [30]. The mixed phases of CeO_2_ and MgO with controlled gel composition during synthesis were characterized by the Rietveld refinement, which quantitatively analyzed the composition ratio of CeO_2_ and MgO as summarized in Table 1 [31,32,33,34].

The resultant as-synthesized products were mostly mixed phases of CeO_2_ and MgO with different compositions, which were used for supporting Au nanoparticles; this was followed by H_2_ treatment for reduction of the supported Au nanoparticles (Figure 1B,C). The initial structure of the CeO_2_ phase in CeO_2_–MgO was almost fully maintained after the Au nanoparticles were supported and subsequently dried (Figure 1B). However, the MgO phase was mostly transformed to a Mg(OH)_2_ phase (*hkl* indices in blue in Figure 1B) as a result of the hydration of MgO in the aqueous solution during the process of supporting the Au nanoparticles. The Au-supporting samples were further treated under H_2_ environment at 300 °C for 5 h to produce metallic Au nanoparticles. After H_2_ treatment, no significant changes were observed in the XRD reflections (Figure 1C). Most of the Mg species was preserved as the crystalline structure of Mg(OH)_2_ (*hkl* indices in blue in Figure 1C) [35], but a small amount of MgO phase developed, as indicated with (200) reflection (*hkl* indices in black in Figure 1C). Any XRD peaks corresponding to the metallic Au nanoparticles were not observed, indicating that the Au nanoparticles were dispersed with a small size distribution and that bulky nanoparticles were not produced.

**Figure 2:**
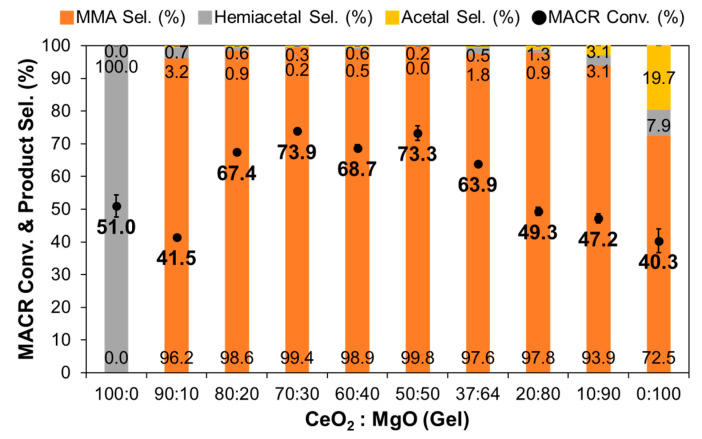
Reaction summaries showing the MACR conversion and product selectivity obtained by using Au/CeO_2_–Mg(OH)_2_ with different Ce:Mg ratios calcined at 750 °C. The results are also summarized in Table 1. The reaction was carried out at 80 °C for 1 h.

**Scheme 1:**
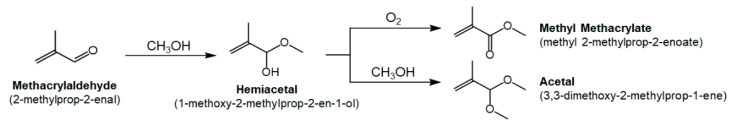
DOE reaction of MACR with methanol under aerobic conditions by using oxygen as the oxidant. MACR can be esterified with methanol to produce a hemiacetal species as the intermediate, which can then be competitively either oxidized to MMA as the desired alkyl ester product or over-esterified with methanol to an acetal species as an undesired byproduct.

The series of Au metal nanoparticles supported on CeO_2_–Mg(OH)_2_ was investigated as the catalyst for the DOE reaction of MACR with methanol (Scheme 1). Figure 2 presents the reaction results and shows the MACR conversion and product selectivity depending on the composition ratio of Ce and Mg in the catalyst support; the results are also summarized in Table 1. The graphs corresponding to the reaction results clearly show the differences in catalytic activity and product selectivity. Au-supporting CeO_2_ (Ce:Mg = 100:0) converted 51.0% of MACR and produced the hemiacetal as the major product with approximately 100% selectivity. The hemiacetal is an intermediate product that can be produced from the first esterification step between MACR and methanol without the subsequent oxidation step (Scheme 1) [7]. Therefore, predominant formation of the hemiacetal indicates that Au-supporting CeO_2_ (Ce:Mg = 100:0) cannot act as a multifunctional catalyst for activating the DOE process.

When Mg(OH)_2_ was introduced to the catalyst support, the Au-supporting CeO_2_–Mg(OH)_2_ catalysts produced MMA as the desired product. As the amount of Mg(OH)_2_ in the CeO_2_–Mg(OH)_2_ support increases, a pseudo-volcano distribution is observed for the MACR conversion (black dots in Figure 2). When the ratio of Ce:Mg was between 80:20 and 37:63 in the synthesis gel, high MACR conversion values 67.4–73.9% were obtained, and the MMA selectivity was close to 100%. When the amount of Mg precursor in the synthesis gel increased beyond 80% (Ce:Mg = 20:80), the MACR conversion dropped below 50%, and the MMA selectivity also gradually decreased. When pure Mg(OH)_2_ was used as the support for Au nanoparticles in the absence of CeO_2_ (Ce:Mg = 0:100), the MACR conversion was 40.3%, and the MMA selectivity was correspondingly reduced to 72.5%. The volcano distribution of MACR conversion and MMA selectivity confirmed that there is an optimum composition for achieving high MACR conversion with MMA selectivity. When the ratio of Ce:Mg was between 80:20 and 37:63 in the synthesis gel, MACR conversion above 65% was achieved with a high MMA selectivity of close to 100% (Figure 2).

### 3.2. Effect of Support Crystallinities in CeO_2_–Mg(OH)_2_ Supporting Au Nanoparticles

**Figure 3:**
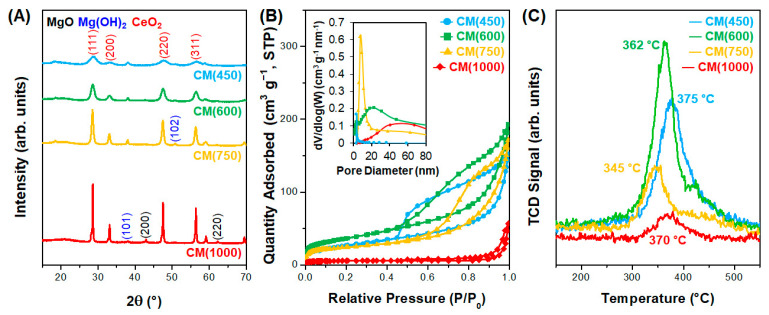
(**A**) X-ray diffraction patterns, (**B**) N_2_ adsorption/desorption isotherms with pore size distributions (inset), and (**C**) CO_2_ temperature-programmed desorption profiles of CeO_2_–Mg(OH)_2_ after calcination at different temperatures: 450 °C (cyan), 600 °C (green), 750 °C (yellow), and 1000 °C (red).

The crystallinities of CeO_2_–Mg(OH)_2_ for supporting Au nanoparticles can be controlled by changing the calcination temperature. From the controlled Ce:Mg ratios, we selected the sample with Ce:Mg = 37:63 as the one showing the optimized catalytic performance (Figure 2). Depending on the calcination temperature, the crystallinities of the CeO_2_–Mg(OH)_2_ supports changed, as evidenced by the XRD patterns shown in Figure 3A. The XRD reflections gradually sharpened as the calcination temperature was increased from 450 to 1000 °C. When the calcination temperature was 450 °C, the crystallinity was significantly lower, as evidenced by very broad XRD reflections. 

The N_2_ adsorption isotherms showed that the CM(600) sample exhibited the highest porosity with a BET surface area (S_BET_) of 128 m^2^ g^−1^ and a total pore volume (V_tot_) of 0.28 cm^3^ g^−1^ (Figure 3B and Table 2). This sample showed a type IV isotherm, which indicates the presence of a mesoporous structure. As the calcination temperature was increased to 750 or 1000 °C, the BET surface area and total pore volume decreased very significantly (Table 2). Among the four samples with different calcination temperatures, CeO_2_–Mg(OH)_2_ calcined at 600 °C (i.e., CM(600)) showed the highest porosity (Table 2). The crystallinities of the CeO_2_–Mg(OH)_2_ samples also affected the surface basicity, as characterized by CO_2_ TPD–MS (Figure 3C). The amounts of total basic sites (B_T_) were proportional to the surface areas determined from the N_2_ adsorption isotherms (Table 2). In terms of basic strengths, the temperatures showing maximum CO_2_ desorption (Figure 3C and T_max_ in Table 2) increased in the following order: CM(750), 345 °C < CM(600), 362 °C < CM(1000), 370 °C < CM(450), 375 °C.

**Figure 4:**
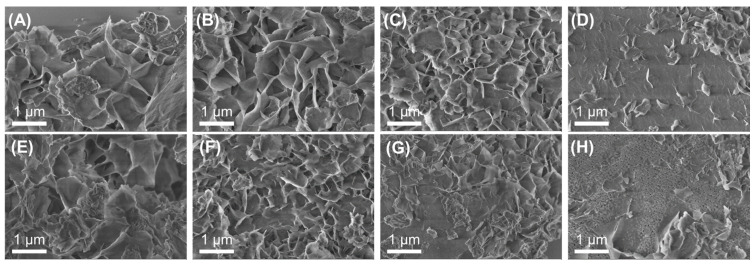
Scanning electron micrograph images of AuCM samples (**A**–**D**) before and (**E**–**H**) after H_2_ treatment: (**A**,**E**) AuCM (450), (**B**,**F**) AuCM (600), (**C**,**G**) AuCM (750), and (**D**,**H**) AuCM (1000).

**Figure 5:**
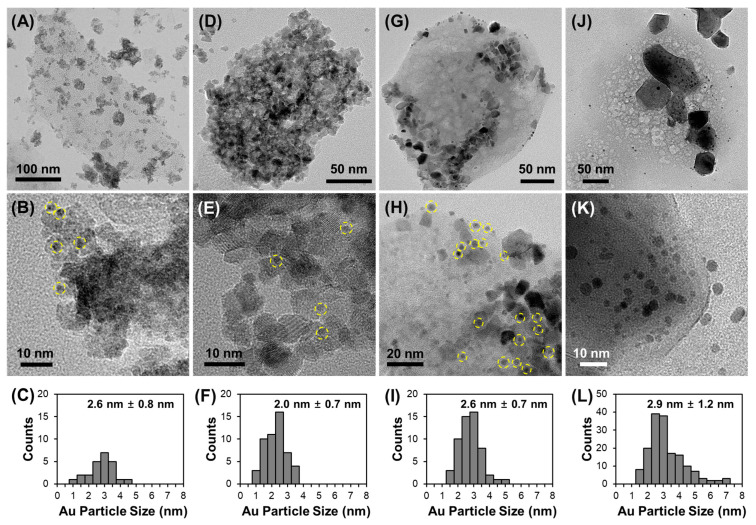
Transmission electron micrograph images and histograms for size distributions of Au nanoparticles of AuCM samples: (**A**–**C**) AuCM(450), (**D**–**F**) AuCM(600), (**G**–**I**) AuCM(750), and (**J**–**L**) AuCM(1000). Yellow dashed circles indicate Au nanoparticles.

Scanning electron microscopy also confirmed that the crystals of the CeO_2_–Mg(OH)_2_ supports were adhered to each other, and the plate-like morphology with a curved shape of CM(450) gradually disappeared as the calcination temperature was increased to 1000 °C (Figure 4A–D). No significant changes in the overall morphologies were observed after H_2_ treatment for the reduction of Au nanoparticles on the CM supports (Figure 4E–H). Control of the calcination temperature changed not only the crystallinity, porosity, and basicity but also the dispersion of the Au nanoparticles and the degree of SMSI at the ternary interface. ICP–OES confirmed that the Au loading is around 2.5–2.7 wt.%, except for AuCM(1000), which supports 1.6 wt.% of Au nanoparticles. Because CM(1000) has the lowest porosity, the Au nanoparticles were supported with a much lower loading. However, regardless of the Au loading, TEM analysis for the Au-supporting CM samples with different calcination temperatures confirmed that the average sizes of the Au nanoparticles were in a similar range (2.0–2.9 nm) (Figure 5 and Table 2). The CM(600) sample supported Au nanoparticles with the smallest average size and the highest dispersion (Table 2).

**Figure 6:**
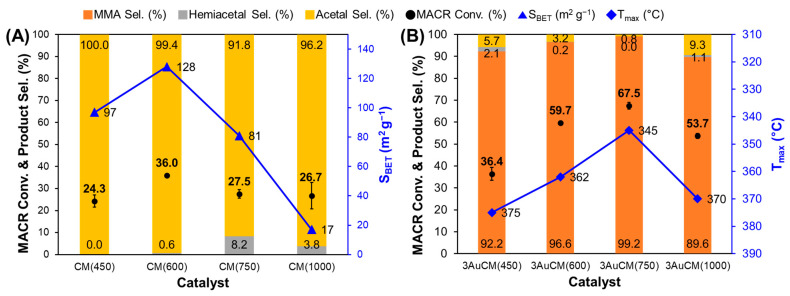
Reaction summaries showing the MACR conversion and product selectivity with CeO_2_–Mg(OH)_2_ in the (**A**) absence or (**B**) presence of Au nanoparticles. The black circles indicate MACR conversion. The orange, gray, and yellow bars correspond to the selectivities of MMA, hemiacetal, and acetal, respectively. The blue guidelines with triangles in (**A**) indicate the BET surface areas (S_BET_) of CeO_2_–Mg(OH)_2_ supports. The blue guidelines with diamonds in (**B**) indicate the maximum desorption temperature (T_max_) of the CeO_2_–Mg(OH)_2_ supports, as determined from the CO_2_ TPD–MS profiles shown in Figure 3C.

The AuCM samples were tested as catalysts for the DOE reaction for comparison with the CM supports without Au nanoparticles. Figure 6A,B show the reaction results obtained by using the series of CM supports and AuCM catalysts, respectively, at 80 °C for 1 h with 9 bar of O_2_, which are summarized in Table 2. Regardless of the calcination temperature, the CM supports not supporting Au nanoparticles failed to activate the DOE process, consisting of esterification and the consecutive oxidation pathway; hence MMA was never produced (Figure 6A). However, the formation of the hemiacetal or acetal proved that the basic surface sites on the CM supports are sufficiently effective to activate the esterification pathway [36,37,38,39,40]. Among the four CM supports, CM(600), with the highest surface area, exhibited the largest MACR conversion of 36.0% (Figure 6A). When Au nanoparticles were supported on the CM supports, MMA was produced as the major product (Figure 6B). This confirmed that the Au nanoparticles are a prerequisite for O_2_ activation to produce MMA via the oxidative esterification pathway (Scheme 1), which is also consistent with Au nanoparticles being efficient oxidation catalysts, as reported elsewhere [7,41,42,43,44,45,46]. Among the four samples, AuCM(750) showed the best performance, achieving the highest MACR conversion (67.5%) and the highest MMA selectivity (99.2%). Centered on AuCM(750), a clear volcano plot was observed. The MACR conversion and MMA selectivity were reduced simultaneously when other catalysts were used (Figure 6B). Notably, the MACR conversion is systematically correlated with the T_max_ value for CO_2_ desorption (Table 2 and blue diamonds in Figure 6B). The number of total basic sites (B_T_) was the highest for AuCM(600), which has the highest BET surface area (S_BET_), but the AuCM(750) catalyst showed the best catalytic performance in terms of MACR conversion and MMA selectivity. The results suggest that basicity with an optimized amount and strength is necessary for the best reaction performance.

The catalytic activity and reaction condition of the AuCM(750) catalyst were compared with that of the other catalysts reported elsewhere (Table 3). Compared to the published works, significant differences were observed in the reaction conditions. For example, we tested our catalyst in the reaction solution with more concentrated MACR (methanol/MACR = 5/1), whereas other studies were tested in a less concentrated MACR condition. Most reactions were carried out at similar temperature of 70–80 °C. Although our catalyst exhibited smaller TON than the others, it should be noted that the AuCM(750) catalyst exhibited the highest MMA selectivity close to 100%.

**Figure 7:**
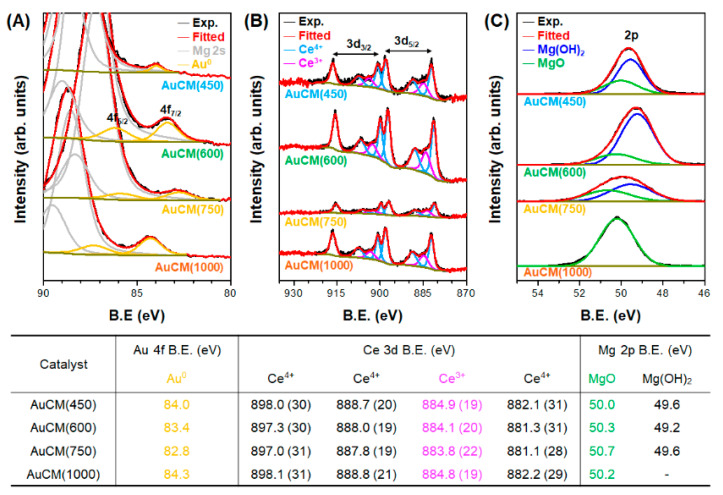
X-ray photoelectron profiles corresponding to the representative binding energy (B.E.) regions of (**A**) Mg 2s, Au^0^, and Au^1+^, (**B**) Ce^4+^ and Ce^3+^, and (**C**) Mg(OH)_2_ and MgO for the series of AuCM catalysts in which the CeO_2_–Mg(OH)_2_ supports were calcined at different temperatures. The table at the bottom summarizes the B.E. values for the representative peaks, and the numbers in parentheses indicate the percentages of elements with different electronic states. The experimental and fitted XPS profiles are drawn in black and red, respectively.

**Figure 8:**
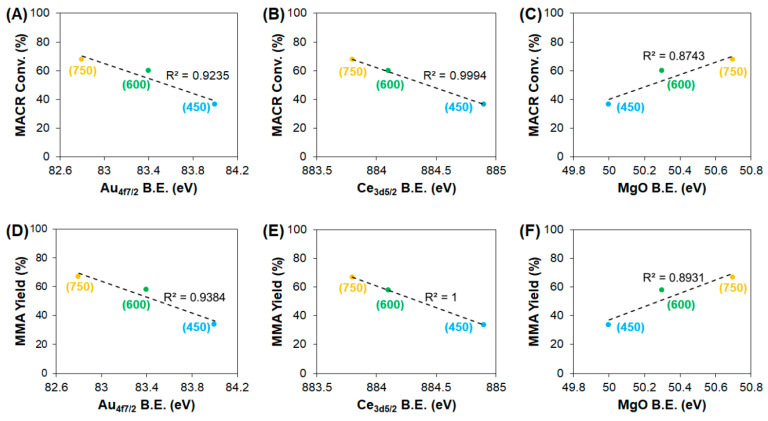
Correlations of (**A**–**C**) MACR conversion or (**D**–**F**) MMA yield with the binding energies of (**A**,**D**) Au 4f_7/2_, (**B**,**E**) Ce 3d_5/2_, (**C**,**F**) MgO, as summarized in the table in Figure 7.

The electronic properties on the surfaces of the ternary components in the AuCM catalysts were further characterized with XPS (Figure 7), and the results were interpreted in relation to the reaction behaviors (Figure 8). The XPS spectra in Figure 7A–C show distinct differences in binding energies for Au 4f_7/2_, Ce 3d_5/2_, and Mg 2p, respectively, depending on the different calcination temperatures of the CM supports. As the calcination temperature increased from 450 to 600 and 750 °C, the binding energies of Au 4f_7/2_ and Ce 3d_5/2_ were shifted to higher values whereas the binding energy of MgO deconvoluted from the Mg 2p peak was shifted to a lower value (table in Figure 7). For the AuCM(1000) sample, the binding energies were shifted with a different trend. Specifically, the XPS signal in the Mg 2p region corresponds to the pure MgO phase without an appreciable deconvoluted peak corresponding to Mg(OH)_2_. As a result of the thermal treatment at the very high temperature of 1000 °C, most of the Mg phase exists as MgO (Figure 7C), which might be the reason for the deviation in the binding energy shift relative to that with the other CM supports calcined at lower temperatures. In addition, the smaller content of Au on the CM(1000) support (1.6 wt.%, Table 2) may be another reason for the different trend in the binding energy shift. The notably weaker intensity of the XPS peaks of AuCM(750) than that of the other samples might be attributed to the fact that the catalyst particles in the AuCM(750) sample were more agglomerated than in the others and hence the electrons that could escape from the surface due to exposure to the XPS incident beam were less concentrated than in the other samples. The TEM image of AuCM(750) sample in Figure 5G is also in line with this explanation, in which the catalytic particles in AuCM(750) look more agglomerated.

The shifts for the binding energies of Au 4f_7/2_, Ce 3d_5/2_, and Mg 2p depending on the CM supports may be evidence of a variation in the SMSI effects among the three components of Au, CeO_2_, and Mg(OH)_2_. As the calcination temperature was changed, the surface characteristics and crystallinities of the CM supports changed. As a consequence, the electronic interactions at the interfaces of Au, CeO_2_, and Mg(OH)_2_ may be different. These different electronic interactions among the catalytic components should affect the catalytic behavior. Indeed, with the exception of the AuCM(1000) sample, the binding energies of Au 4f_7/2_, Ce 3d_5/2_, and MgO in the AuCM(450, 600, 750) samples were related to the MACR conversion and MMA yield (Figure 8). The graphs clearly show very linear relationships between the reaction behaviors and the binding energies. As the binding energies of Au 4f_7/2_, and Ce 3d_5/2_ increased, the MACR conversion and MMA yield decreased in a very linear manner. By contrast, as the binding energy of MgO increased, the MACR conversion and MMA yield also increased in a linear manner. The correlations appear very linear, as confirmed by the R^2^ values close to 1 (Figure 8), and such linear correlations suggest that the different SMSI effects between the metal nanoparticles and the supporting materials may contribute to the catalytic performances.

## 4. Conclusions

The effect of the supporting material for Au nanoparticles on the catalytic behavior during the DOE reaction of MACR with methanol to selectively produce MMA as the desired product has been investigated by controlling the Ce:Mg ratios and crystallinities in the CeO_2_–Mg(OH)_2_ supports. The reaction studies revealed that there was an optimum Ce:Mg ratio and crystallinity for achieving the highest MACR conversion and MMA selectivity with Au-supporting CeO_2_–Mg(OH)_2_ as a multifunctional catalyst. The sole presence of CeO_2_ or Mg(OH)_2_ for supporting the Au nanoparticles is not efficient for the DOE reaction consisting of oxidation and esterification pathways. Optimum catalytic activity of the CeO_2_–Mg(OH)_2_ support was obtained when the support was calcined at 750 °C; the AuCM(750) catalyst showed significant shifts of the binding energies for Au 4f_7/2_ and Ce 3d_5/2_ to the lowest values, whereas the binding energy of MgO deconvoluted from the Mg 2p peak in AuCM(750) was shifted to the highest value. As a result, AuCM(750) with a Ce:Mg ratio of 37:63 exhibited the highest MACR conversion of 67.5% with the highest MMA yield of 67.0% within 1 h of reaction at 80 °C. The significant enhancement in the catalytic activity of the AuCM catalyst can be attributed to the different SMSI effects among the three catalytic components of Au, CeO_2_, and Mg(OH)_2_; this effect can be controlled by the composition of CeO_2_ and Mg(OH)_2_ and by the calcination temperature of the CeO_2_–Mg(OH)_2_ support. The catalytic studies described herein can provide an important method for controlling SMSI effects and, thereby, the catalytic behaviors of multifunctional heterogeneous catalysts.

## Figures and Tables

**Table 1:** Reaction summaries showing the MACR conversion and product selectivity by using the Au/CeO_2_–Mg(OH)_2_ catalysts with different Ce:Mg ratios calcined at 750 °C. ^[a]^ These results are also graphically represented in Figure 2.

Ce:Mg Ratio				MACR Conv. (%)	MMA Sel. (%)	Hemiacetal Sel. (%)	Acetal Sel. (%)	Y_MMA_ (%) ^[b]^
Gel		Rietveld						
Ce	Mg	CeO_2_	MgO					
100	0	100	0	51.0	0.0	100.0	0.0	0.0
90	10	98	2	41.5	96.2	3.2	0.7	39.9
80	20	97	3	67.4	98.6	0.9	0.6	66.5
70	30	92	8	73.9	99.4	0.2	0.3	73.5
60	40	85	16	59.9	98.8	0.0	1.2	59.2
50	50	79	21	73.3	99.8	0.0	0.2	73.2
37	63	73	27	67.5	99.2	0.0	0.8	67.0
20	80	48	52	49.3	97.8	0.9	1.3	48.2
10	90	26	74	47.2	93.9	3.1	3.1	43.3
0	100	0	100	40.3	72.5	7.9	19.7	29.2

^[a]^ General reaction conditions: methanol/MACR = 5/1, MACR 0.0653 mol, O_2_ pressure of 9 bar at 80 °C for 1 h; ^[b]^ Y_MMA_: yield of MMA calculated as (MACR conv. × MMA sel./100).

**Table 2:** Physicochemical properties of AuCM catalysts with different calcination temperatures of 450, 600, 750, and 1000 °C and their catalytic reaction summaries showing the MACR conversion and product selectivity ^[a]^.

Catalyst	Au Content (wt.%) ^[b]^	Au Size (nm) ^[c]^	Au Dispersion (%) ^[d]^	S_BET_ (m^2^ g^−1^) ^[e]^	D_p_ (nm) ^[f]^	V_tot_ (cm^3^ g^−1^) ^[g]^	B_T_ (mmol g^−1^) ^[h]^	T_max_ (°C) ^[i]^	MACR Conv. (%)	MMA Sel. (%)	Hemiacetal Sel. (%)	Acetal Sel. (%)	Y_MMA_ (%) ^[j]^	TON ^[k]^	STY ^[l]^
AuCM (450)	2.7	2.6	45.0	97	3.3	0.23	0.32	375	36.4	92.2	2.1	5.7	33.6	413	381
AuCM (600)	2.7	2.0	58.4	128	5.4	0.28	0.45	362	59.7	96.6	0.2	3.2	57.7	264	255
AuCM (750)	2.5	2.6	44.4	81	7.4	0.25	0.24	345	67.5	99.2	0.0	0.8	67.0	521	517
AuCM (1000)	1.6	2.9	40.3	17	40.3	0.07	0.07	370	53.7	89.6	1.1	9.2	48.1	714	640

^[a]^ General reaction conditions: methanol/MACR = 5/1, MACR 0.0653 mol, O_2_ pressure of 9 bar at 80 °C for 1 h; ^[b]^ determined by ICP–OES; ^[c]^ determined from TEM images; ^[d]^ determined by mean size of Au particles; ^[e]^ S_BET_: BET surface area determined by the BET method; ^[f]^ D_p_: average pore diameter determined by the BJH method; ^[g]^ V_tot_: total pore volume determined by the BJH method; ^[h]^ B_T_: total amount of basic sites determined by CO_2_ TPD–MS; ^[i]^ T_max_: desorption temperature showing the maximum intensity in the CO_2_ TPD–MS profiles; ^[j]^ Y_MMA_: yield of MMA calculated as (MACR conv. × MMA sel./100); ^[k]^ TON (mol_MACR_ mol_Au_^−1^): turnover number calculated as (moles of MACR reacted/moles of active Au); ^[l]^ STY (mol_MMA_ mol_Au_^−1^ h^−1^): site–time yield calculated as (moles of MMA produced/moles of active Au/time).

**Table 3:** Catalytic activity and reaction condition of AuCM(750) catalyst in comparison with the others reported in many literatures.

Catalyst	Quantity (g)	Methanol/MACR	Temp. (°C)	Pressure (MPa)	Time (h)	MACR Conv. (%)	MMA Sel. (%)	TON	Ref.
AuCM(750)	1.5	5/1	80	0.9	1	67.5	99.2	521	This work
Au–CeO_2_/ɤ–Al_2_O_3_	0.5	20/1	70	0.2	2	97	90	-	[24]
Au/Ce_x_Zr_y_O_z_	0.5	20/1	80	0.3	2	99	74	577	[25]
Pd_3_Pb	2.5	8/1	80	0.3	2	89	79	-	[26]
Au/ZnO	0.5	30/1	70	0.2	2	99.9	85.9	-	[36]
Au/La_2_O_3_	0.1	8/1	70	0.2	2	89	98	1136	[39]
